# Listening to the community: identifying obesity prevention strategies for rural preschool-aged children

**DOI:** 10.3389/fpubh.2024.1372890

**Published:** 2024-05-31

**Authors:** Katherine Jochim Pope, Alexandra F. Lightfoot, Lisa Macon Harrison, Deborah Getz, Joel Gittelsohn, Dianne Ward, Tamara S. Hannon, Temitope Erinosho

**Affiliations:** ^1^Department of Applied Health Science, Indiana University Bloomington, Bloomington, IN, United States; ^2^Department of Health Behavior, North Carolina Translational and Clinical Sciences Institute, University of North Carolina at Chapel Hill, Chapel Hill, NC, United States; ^3^Granville Vance Public Health Department, Henderson, NC, United States; ^4^Department of International Health, Johns Hopkins University, Baltimore, MD, United States; ^5^Department of Nutrition, University of North Carolina at Chapel Hill, Chapel Hill, NC, United States; ^6^Department of Pediatrics, Indiana University School of Medicine, Indianapolis, IN, United States

**Keywords:** childhood obesity, multi-level interventions, community engagement, rural, preschool-aged children

## Abstract

Multi-level interventions promoting healthy weight in rural preschool children aged 2–5 years are limited. With the goal of developing a community-informed obesity prevention intervention for rural preschool-aged children, the purpose of this descriptive study was to identify: (1) community settings and intervention strategies to prioritize for an intervention; (2) potential implementation challenges and solutions; and (3) immediate interventions the study team and community partners could collaboratively implement. Workshops occurred in two rural communities in Indiana (2 workshops) and North Carolina (2 workshops), with high obesity rates. A guide was developed to moderate discussions and participants voted to rank community settings and intervention strategies. There were 9–15 participants per workshop, including parents, childcare providers, and representatives of community organizations. Community settings identified as priorities for child obesity prevention included the home, educational settings (preschools), food outlets, recreational facilities, and social media. Priority intervention strategies included providing nutrition and physical activity education, increasing access to healthy foods and physical activity in the built environment, and enhancing food security. Potential intervention implementation challenges centered on poor parental engagement; using personalized invitations and providing transportation support to families were proffered solutions. Immediate interventions to collaboratively implement focused on making playgrounds esthetically pleasing for physical activity using game stencils, and nutrition education for families via quarterly newsletters. This participatory approach with community partners provided insight into two rural communities’ needs for child obesity prevention, community assets (settings) to leverage, and potential intervention strategies to prioritize. Findings will guide the development of a multi-level community-based intervention.

## 1 Introduction

Childhood obesity rates in the United States (U.S.) are high. From 2010 to 2020, obesity prevalence in children aged 2–5 years increased from 10 to 13% (1). This public health concern is more acute in rural communities, with studies reporting 26% higher odds of obesity in rural versus urban children (2). Obesity prevention is preferrable to treatment in rural children (3), but often difficult to achieve because of multiple risk factors (4, 5) occurring at the child (e.g., child diet/physical activity [PA]), family (e.g., socioeconomics), organizational (out-of-home care settings), community (e.g., built environment), and policy levels (6, 7). Interventions targeting a single level of influence demonstrate mixed results in terms of effects on child weight (8). To effectively address child obesity in rural areas requires that interventions simultaneously target multi-level influences. The Socioecological Model, which posits that child obesity is influenced by factors at multiple levels of influence, including individual, interpersonal (family), organizational, community, and policy levels (9), provides a framework for understanding the critical need for multi-level child obesity prevention interventions.

Multi-level community-based interventions (e.g., Shape Up Somerville, Romp & Chomp) have been shown to promote sustainable improvements in child weight (10–16). This type of intervention exposes entire communities to obesity prevention efforts and simultaneously targets change at multiple levels that influence child obesity (e.g., child and family) (16, 17). Applying this intervention approach requires that researchers engage with persons having first-hand knowledge about communities to ensure applicability, effectiveness, and sustainability of an intervention (18, 19). To the authors’ knowledge, there is one multi-level, childhood obesity prevention intervention that has targeted rural U.S. communities, with results unpublished (20, 21), but no such studies have targeted rural children aged 2–5 years.

Although rural communities have strengths, including the tightknit social ties among residents, strong cultural traditions, and proximity to natural landscapes that offer opportunities for outdoor activities (22–25), lack of access to resources that support wellbeing can make it difficult to implement and sustain interventions in rural communities. From June 2019 to July 2021, the current study team conducted formative research in two rural communities in Indiana (IN) and North Carolina (NC) to identify barriers, facilitators, and opportunities to address obesity in preschool children aged 2–5 years (published elsewhere) (26). Guided by the formative research, with the goal of developing a community-based intervention for preschool-aged children, the study team conducted workshops to engage with partners from the two rural communities (e.g., parents, representatives of community organizations) in the identification of: (1) community settings to prioritize for a child obesity prevention intervention; (2) intervention strategies at multiple levels of influence (e.g., child, family) to prioritize; (3) challenges that might be encountered while implementing an intervention, with potential strategies for navigating challenges; and (4) immediate interventions the study team and community partners could begin to implement collaboratively with little or no funding. This paper describes results from the workshops.

## 2 Methods

### 2.1 Study setting and participants

This descriptive study occurred in spring of 2022 in two rural counties (“communities” hereon) in IN and NC. Rurality was defined using U.S. Department of Agriculture’s Rural–Urban Commuting Area Codes (27). Both communities are considered high-need, with child poverty levels (18–32%) (28, 29) that exceed the national poverty average (16%) (28, 30), and high child and/or adult obesity (20–39%). Both communities differ in racial/ethnic make-up; the IN community is predominantly (96%) non-Hispanic White (31), while the NC community is diverse, with Black/African-Americans comprising 52% and Hispanic/Latino, 9% (32). Study participants included parents of children aged 2–5 years, childcare providers, representatives of community organizations serving children/families, and community residents interested in improving child health.

To recruit participants, two study team members (KP, TE) participated in a meeting for an existing coalition of community leaders in each community. At each meeting, the study team shared initial results from the formative research conducted to learn about barriers, facilitators and opportunities to promote healthy weight in children aged 2–5 years in both communities (26). The study team invited coalition members to participate in community workshops, sought insight from coalition members about how to structure the workshops (e.g., where/when to host workshops, incentives to offer), and enlisted their assistance with participant recruitment. Thereafter, personalized invitations were sent to coalition members, other community leaders who were not members of the coalitions (e.g., librarians, faith-based leaders), and persons from the formative research (26). Coalition members and other community leaders received several copies of the invitation to distribute to community residents in their network. Persons interested in participating in the workshops were instructed to notify the study team by telephone/email.

Overall, 110 invitation cards (65 in IN, 45 in NC) were mailed, with the goal to recruit up to 15 participants per community, a threshold that would allow for robust discussions among participants based on the study team’s prior experiences with conducting community workshops (33, 34). The study team aimed to recruit a diverse representation of participants, including parents, childcare providers, and representatives of community organizations that serve families, but there were no set quota requirements. Two workshops were held per community. In IN, 15 persons participated in the first workshop, while 11 participated in the second workshop. In NC, there were 9 persons in the first workshop, and 13 in the second workshop. Study procedures were approved by the Institutional Review Board at Indiana University Bloomington. Written informed consent was obtained from participants before each workshop.

### 2.2 Overview of the community workshops

Community workshops occurred on Saturday mornings at publicly accessible community facilities. Short breaks were incorporated, refreshments were provided, and participants received a thank-you gift. The first workshop was 3 h and participants received $75 upon completion, versus $50 for the second workshop lasting 2 h. The study team developed a discussion guide (Supplementary Table 1) for the workshops that was informed by the formative research in the two communities (26) and similar studies that used community workshops to design community-based interventions (18, 33, 34). The Socioecological Model (9) and previous multi-level obesity prevention studies (10, 11, 13, 18, 35, 36) provided a theoretical framework to understand influencing factors, and prioritized community settings and strategies to promote healthy dietary intake and PA at the child, family/peer, organizational (e.g., childcare settings), community (e.g., built environment), and policy levels. The workshops were intended to be interactive and participatory. Each workshop began with a description of the purpose of the workshop, completion of informed consent and a demographic survey by participants, and an ice-breaker activity. Facilitation of each workshop was led by the same study team member (TE), with assistance from another team member (KP/AL). Flip charts displayed in the meeting room were used to record participants’ responses, and discussions were audio-taped.

### 2.3 Data collection at the community workshops

**Figure 1:**
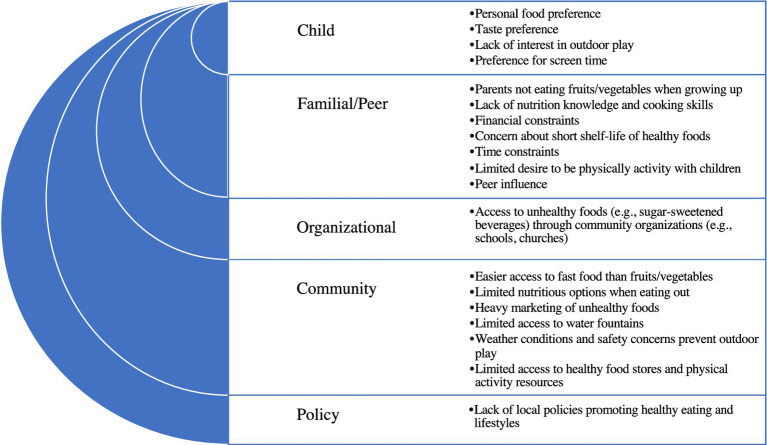
Factors influencing the consumption of unhealthy foods and physical inactivity among families with children aged 2–5 years in two rural communities in Indiana and North Carolina: summary of results from community workshops.

Guided by prior childhood obesity prevention studies (11–15), data collection for this study focused on two behavioral targets, to: (i) promote healthy dietary intake (specifically, increase fruits and vegetables; reduce fast food; reduce sweet/salty snacks; reduce sugar-sweetened beverages; and promote water consumption) and (ii) promote PA (Figure 1). Similar contents were covered at each workshop across both communities using the discussion guide, however, where necessary, the facilitator combined behavioral targets for discussions because of time constraints. Data collection began with a discussion among participants about factors influencing the choice to engage in the target dietary and PA behaviors in families with children aged 2–5 years in various settings (home, childcare). While related information was collected in the formative research, discussions around influencing factors that impact child healthy weight behaviors helped to set the stage for ensuing discussions about settings and strategies to prioritize in an intervention. Participants’ responses were recorded on flip charts and then reflected back at the end of the discussion.

Next, participants were asked to specify community settings in which to intervene to promote each behavioral target. After this discussion was exhausted, four sticky dots were provided to each participant so they could vote on settings they thought should be prioritized in an intervention. Votes were tallied and reported back to participants. During the next phase of discussions, participants were asked to specify potential intervention strategies for each behavioral target. Given time constraints, some behavioral targets were combined for discussion (e.g., strategies to reduce sugar-sweetened beverages combined with strategies to promote water consumption). After the discussion was exhausted, participants received 4 to 6 sticky dots to vote on intervention strategies that they thought should be prioritized for each behavioral target. Votes were tallied and restated to participants. The moderators then asked participants to specify challenges they thought the study team might encounter in implementing a potential intervention and strategies that might help to navigate challenges. Given time constraints in IN, the discussion about challenges occurred only in NC, and participants’ responses were captured on flip charts.

Final discussions centered on identifying immediate interventions from the priority list that the study team and community partners could begin to work on with little or no funding, and how the community and academic partners could begin to work together collaboratively to develop a multi-level obesity prevention intervention for children aged 2–5 years and families in their community. The study team shared workshop summaries with participants by email about 10 days after each workshop and also provided a printed copy of the summary from the first workshop at the second workshop. Email and in-person communications included requests for participants to check that the summaries accurately reflected discussions held, and report related concerns.

### 2.4 Data analysis

Audio-taped recordings from each workshop were transcribed without identifiers and were reviewed for accuracy and completeness. Data coding and content analysis were conducted by study team members trained in qualitative analysis (TE, KP) using the transcripts, supplemented with flipchart notes. Differences in the application of codes and content analyses were discussed by the coders and resolved by consensus. Notably, participants’ responses about factors that influence the choice to engage in the target dietary and PA behaviors were coded into five descriptive categories, guided by the Socioecological Model (9) and prior multilevel child obesity prevention research (10, 11, 13, 18, 35, 36): child; family; organizational; community; and policy. Community settings with the four highest votes for each behavioral target were coded into descriptive categories based on similarities in function (e.g., educational, recreational settings) by state. A similar process was used to code and summarize responses about potential intervention strategies to prioritize. Responses about challenges the study team might encounter in implementing a potential intervention, strategies for navigating challenges, and immediate interventions to begin to implement were described. Demographic characteristics of participants were summarized using frequencies and percentages in R (version 4.2.1, Vienna, Austria), a software for quantitative analyses.

## 3 Results

Demographic characteristics of participants are shown in Table 1. There were 9–15 participants per workshop. The second workshop included some of the persons who had participated in the first workshop (5 in IN, 7 in NC) and new participants (5 in IN, 6 in NC). Participants described themselves as parents, grandparents, child-care providers, representatives of community organizations (e.g., healthcare, business, government, youth service), or a combination of those roles. Participants were predominantly female, with more racial diversity in NC compared to IN.

**Table 1:** Demographic characteristics of participants in community workshops conducted in two rural communities in Indiana and North Carolina^1^.

	Indiana community		North Carolina community	
	Workshop 1 (*n* = 15)	Workshop 2 (*n* = 11)	Workshop 1 (*n* = 9)	Workshop 2 (*n* = 13)
	*n* (%)	*n* (%)	*n* (%)	*n* (%)
*Sex*				
Female	14 (93)	10 (91)	8 (89)	10 (77)
Male	1 (7)	1 (9)	1 (11)	3 (23)
Age				
18–35 years	4 (27)	2 (18)	3 (33)	5 (38)
36–55 years	7 (47)	6 (55)	4 (44)	4 (31)
55 years or older	4 (27)	3 (27)	2 (22)	4 (31)
*Race*				
Black/African-American	0 (0)	0 (0)	4 (44)	10 (77)
White	13 (87)	11 (100)	3 (33)	3 (23)
Other	2 (13)	0 (0)	2 (22)	0 (0)
Ethnicity				
Hispanic	1 (7)	0 (0)	0 (0)	0 (0)
Non-Hispanic	14 (93)	11 (100)	9 (100)	12 (100)
Employment status				
Full-or part-time	12 (80)	9 (82)	7 (78)	9 (69)
Not currently employed	3 (20)	2 (18)	2 (22)	4 (31)
*Self-identified roles* *^2^*				
Parent	5	3	2	4
Grandparent	1	0	1	2
Childcare provider	3	2	0	1
Youth Service	2	3	3	3
School	4	2	0	0
Business/Media	1	0	2	1
Law enforcement/ government	0	0	1	1
Civic, volunteer, or religious	3	2	2	2
Healthcare	3	1	2	3
Other	0	0	0	2

1 The total number of participants in each workshop across the Indiana and North Carolina community were not calculated in this table because some participants took part in both workshops, while others participated in a single workshop. To preserve participant anonymity, the study team asked each participant to complete a demographic survey at the start of each workshop, even if they had participated in the previous workshop. Thus, the study team was unable to differentiate demographic characteristics of persons who participated in a single workshop versus both workshops.

2 When reporting self-identified roles, participants could “select all that apply.” Therefore, this table is not indicative of the total count of participants, but rather, the roles that were represented at the workshops., hence the reason for not combining categories or including percent counts.

Participants listed examples of factors that influence the choice to engage in the target dietary and PA behaviors but did not prioritize or rank factors in order of importance (Figure 1). At the child level, these included child preference for unhealthy foods, peer influence, lack of interest in outdoor play, and preference for electronic media. A participant said: “*I really want her [child] to eat healthy, but at the same time, I want her to eat. She’s literally… her food choices, she will eat chicken nuggets. She will eat French fries. She will eat ramen, carrots, and grapes. That’s it.”* Familial and peer influences included parental perception that healthy foods are expensive with short shelf-life, limited knowledge about how to obtain, prepare or preserve fruits/vegetables, and lack of time to prepare nutritious meals and/or be active with children. A participant described: *“I think it’s the balance, but I’m really blessed to have the life where I can do this. We are very intentional when we eat at home. Everything is healthy at home…when we go to grandparents, that’s kind of the time for the treat… Not everyone has that option.”* Organizational factors centered around perceptions that unhealthy foods were easily accessible through community organizations (e.g., schools, churches).

Community influences included lack of access to outlets that carry healthy foods (supermarkets) and resources that promote PA (parks), marketing of unhealthy foods to children, outdoor weather, and concern about child safety while playing outdoors. Describing the lack of access to PA resources, a participant said: “*One really sad thing that happened with the little kid basketball program was they used to have a preschool and kindergarten little boys’ basketball, biddy ball. Then this year, because it had to get serious, you had to try out as a first grader. Kindergarten and preschool was dropped.”* Additionally, lack of policy to support healthy eating and PA was cited as a challenge. Describing this, a participant said: *I do think there’s a role for the community as a whole. And that would be the government to make the rules of how we are exposed, to make our decisions… If we recognize there’s a problem, there’s only one way that we can step forward as a community to do that: to set some principles and rules that guide us in that.”*

Community settings that participants ranked highest as the top places to promote healthy dietary intake and PA in children are described in Table 2. For the promotion of healthy dietary intake, settings that overlapped between the IN and NC communities included educational settings (e.g., childcare centers), food outlets (e.g., grocery stores), youth sports, community gathering places (e.g., churches), and social media. Also, participants prioritized the home (NC), recreation facilities (IN), and other locations (e.g., community events) (NC) as settings to promote healthy dietary intake in their communities. In terms of promoting PA, settings that overlapped across both communities were educational and recreation facilities (e.g., parks, trails). Additional settings for promoting PA that participants prioritized were the home (NC), social media (IN), and community gathering places (IN).

**Table 2:** Community settings that workshop participants in the two rural communities in Indiana and North Carolina ranked highest for the promotion of healthy dietary intake and physical activity in children aged 2–5 years.

	Indiana community			North Carolina Community		
	Settings that ranked as top 4 places to promote healthy eating and PA			Settings that ranked as top 4 places to promote healthy eating and PA		
Setting to promote:	Setting category	Specific setting	# of votes	Setting category	Specific setting	# of votes
Fruits/vegetables	Educational	Preschools	10	Educational	Child care centers	7	Youth sports	Concession stands	10	Home	Home	6	Social media	Social media	6	Social media	Social media	5	Food/beverage outlets	Farmers’ markets	6	Food/beverage outlets	Grocery stores	4	Food/beverage outlets	Grocery stores	6	Community events	Community events	4							
Less fast food	Youth sports	Concession stands	14	Educational	Child care centers	7	Where parents are	Where parents are	13	Community gathering	Library	5	Recreational	Parks	10	Community gathering	Churches	4	Educational	Schools/cafeteria	5	Food/beverage outlets	Grocery stores	4							
Less sweet/salty snacks	Recreational	Pools	15	Educational	Schools	7	Youth sports	Concession stands	14	Other	Afterschool	7	Educational	Schools	14	Home	Home	6	Recreational	Parks	7	Youth sports	Youth sports	4
Healthy beverages^1^	Food/beverage outlets	Water fountains	10				Recreational	Parks	8	Educational	Schools/cafeteria	8	Educational	Preschools	8
Physical activity	Community gathering	Library	12	Educational	Child care centers	8	Educational	Schools/preschools	8	Educational	Schools	8	Social media	Social media	4	Home	Home	7	Recreational	Walking trails	4	Recreational	Recreation centers	6	Community gathering	Churches	4			

PA represents physical activity.

1 Given time constraints, discussions about setting to promote less consumption of sweet and salty snacks, and healthy beverages were combined at the workshop in the North Carolina community.

Intervention strategies that participants ranked highest are shown in Table 3. Strategies for promoting healthy dietary intake overlapped between the two communities, focusing on: providing nutrition education opportunities (e.g., nutrition education for parents, fruit and vegetable gardening with children at preschools); enhancing access to healthy foods in the built environment (e.g., via community gardens); and enhancing food security through access to food programs (e.g., backpack buddy programs at childcare settings to provide children from food-insecure households with take-home meals). Partnerships with community organizations to increase healthy food offerings in childcare settings were also recommended (NC). For PA promotion, an intervention strategy that overlapped between both communities centered on providing PA education opportunities for children/families (e.g., PA lessons at childcare, organized community events that promote PA). Additional strategies that participants prioritized included: providing enhanced access to PA-promoting resources in the built environment (e.g., adding game stencils to playgrounds) (IN); offering incentives (e.g., free passes to bounce houses) (IN); and leveraging community facilities and local organizations to offer PA to families (NC).

**Table 3:** Intervention strategies that workshop participants in the two rural communities in Indiana and North Carolina ranked highest for the promotion of healthy dietary intake and physical activity in children aged 2–5 years.

	Indiana community			North Carolina community		
	Intervention strategies that ranked as top 4 ways to promote healthy eating and PA			Intervention strategies that ranked as top 4 ways to promote healthy eating and PA		
Ways to promote:	Intervention Category	Specific Intervention	# of votes	Intervention Category	Specific Intervention	# of votes
Fruits/Vegetables^1^	Nutrition education	Hands-on gardening	12	Nutrition education	Nutrition education	8	Enhance access in the built environment	Turn on water fountains (COVID-19 related)	7	Enhance food security	Backpack buddy programs	7	Nutrition education	Provide seeds to kids to grow	6	Enhance access in the built environment	Community gardens	5	Enhance food security	Provide meals kits	6	Nutrition education	Farm tour/field trips	5
						
Less Fast Food				Enhance food security	Grocery store coupons	8	Enhance food security	“Pay as you can” at farmers’ markets	7	Nutrition education	Sampling of foods at grocery stores	6	Enhance food security	Gift cards to farmers’ markets	6
Less sweet/salty snacks andSugary beverages	Enhance access in the built environment	Pop-up fruit & vegetable markets	13	Nutrition education	Community events	10	Nutrition education	Promote healthy celebration foods	10	Community partnerships	Partnerships to increase healthy offerings	10
Physical activity	Enhance access to PA in the built environment	Install game stencils at community locations	11	PA education	Use local personal trainers	8
	PA education	PA lesson in preschool	7	Community partnerships	Use organization to bring in activities	8
	Enhance access to PA in the built environment	Library of “things” to increase PA	7	PA education	Yoga for kids	7
	Provide incentives	Passes to PA locations	6	PA education	Community PA events and classes	6
	PA education	Use social media to promote PA	6			

PA represents physical activity.

1 Given time constraints, discussions about intervention strategies centered around healthy foods in the Indiana community, as opposed to focusing on specific nutrition behavioral targets as was done in the North Carolina community.

Due to time constraints, discussions about challenges the study team might encounter in implementing an intervention and potential navigation strategies occurred only in NC. The major challenge that was discussed centered around low parental engagement in an intervention that might occur because of parents’ busy schedules (lack of time) and limitations with transportation given the community’s lack of a public transit system. Participants suggested using personalized invitations to enhance parental engagement. Organizing intervention activities to occur at community settings where parents typically spend time with children (e.g., childcare centers, parks) was also suggested. Another concern that was discussed centered on the transience of community partners and health initiatives that made it difficult to create sustainable health promotion programs, but no solutions were proffered.

Participants identified immediate interventions they could begin to implement with the study team with limited funding. In IN, the immediate intervention was to install game stencils at public playgrounds/parks to promote PA in children, whereas in NC it was to create a quarterly newsletter about healthy lifestyles to disseminate to families. Participants shared examples of local agencies [e.g., REMC Electric Company (IN), Triangle North Healthcare Foundation (NC)] from which grant funding could be sought to support the immediate interventions. Participants indicated willingness to continue to engage with the study team via quarterly meetings to advance the obesity prevention efforts identified from this study.

## 4 Discussion

This paper describes results from four workshops with community partners to guide the development of a rural multi-level community-based intervention to promote healthy weight in children aged 2–5 years. In the current study, participants described factors influencing the choice to engage in healthy weight behaviors in their community. They cited several factors at the child (e.g., child preference), familial/peer (e.g., financial and time constraints), organizational (e.g., limited access to healthy foods and PA opportunities through organizations), community (e.g., food deserts), and policy levels (lack of nutrition and PA-promoting policies). Participants’ responses about factors that influence the choice to eat healthy and be physically active were consistent with the initial formative research conducted by the current study team in both communities (26) and other studies of rural communities (4, 7, 37).

Discussions at the workshops were used to identify community settings to prioritize in a rural, obesity prevention intervention for children aged 2–5 years. Rural areas vary widely with regards to the availability of resources that can support healthy lifestyles (37) (e.g., supermarkets, recreation centers), but existing community-identified settings can serve as trusted, anchor organizations that can be leveraged in the implementation of community-based child obesity prevention interventions (37, 38). Community settings that participants identified align with studies of children and adults that report social media, rural social networks (e.g., social or family gatherings) (10, 11, 18, 37), food outlets (10, 11, 18, 35–37, 39), and shared community spaces (e.g., schools, faith-based/civic organizations) (10, 11, 13, 18, 37, 40) as natural settings to reach and engage with rural children/families. Notably, representatives of healthcare organizations were present at the workshops and discussions about services/programs available at healthcare settings in the community occurred, however, participants did not prioritize healthcare settings as places to reach or intervene with families.

Intervention strategies identified by study participants can be implemented across several of the settings they prioritized. Providing nutrition and PA education opportunities to children/families and offering incentives to promote healthy lifestyles were recommended by participants. Given the paucity of nutrition and PA resources in most rural areas (26), it not a surprise that participants recommended the need to increase access to healthy foods and PA-promoting resources in their community’s built environment. With many rural areas’ high levels of food insecurity (41), it is also not a surprise that participants recommended enhancing food security in their community through access to food programs. Going forward, the goal of the study team is to work collaboratively with community partners to develop a multi-level intervention that incorporates the community-identified priorities for obesity prevention for children aged 2–5 years, and then seek grant funding to pilot-test the intervention.

Using a community-engaged approach, as was done in the current study, helps researchers build trust with partners in rural communities (18, 37, 42, 43) and allows researchers and community partners to work together in a collaborative manner to design child obesity prevention interventions that are culturally-appropriate, relevant, and acceptable to communities (18, 34, 42). This community-engaged approach is crucial for creating community-based interventions that are likely to be impactful and sustainable in the long-term (18, 38, 44).

At the workshops, participants discussed the installation of game stencils at public playgrounds/parks to promote PA in children (IN) and the dissemination of a quarterly newsletter about healthy lifestyles to families (NC) as immediate interventions that could be implemented with limited funding. To implement these, in IN, the study team collaborated with a community partner (Greene County Foundation, IN) to apply for two small grants that were awarded in the fall of 2022 by the South Center Indiana REMC and the Bloomington Board of Realtors. Using the grant funds, the study team and community partners have painted playground stencils for use by children at three public libraries and two childcare centers. For NC, the study team is working with community partners to develop a series of electronic newsletters, the first of which was shared with community partners in the spring of 2023 to distribute to families served through their respective organizations’ communication channels.

This study has some limitations. Because rural areas differ with regards to resources available to promote healthy weight behaviors, the findings of this study may not be generalizable to all rural communities. While the study team spread the word about the workshops throughout the communities, it is possible that the sample was biased toward persons most interested/passionate in promoting health in their community. Additionally, workshops occurred in the main townships of both counties, thus, excluding participation by interested community members without access to a means of transportation. Childcare support was not provided at the workshops, limiting attendance by parents who could not afford or find childcare. Nevertheless, a strength of this study is the sizable number of participants (9–15) with varied demographic characteristics that allowed for the inclusion of diverse perspectives at the workshops. Additionally, the use of a participatory approach in which community partners and the study team collaboratively identified community priorities for preventing obesity in children aged 2–5 years is a strength.

Results from each workshop were summarized and shared with participants and other community partners via a factsheet. The study team will use the results to work collaboratively with community partners to develop a rural multi-level community-based obesity prevention intervention for children aged 2–5 years.

## Data availability statement

The raw data supporting the conclusions of this article will be made available by the authors, without undue reservation.

## Ethics statement

The studies involving humans were approved by Human Research Protection Program (HRPP) Office for Research Compliance Indiana University Bloomington. The studies were conducted in accordance with the local legislation and institutional requirements. The participants provided their written informed consent to participate in this study.

## Author contributions

KP: Writing – review & editing, Writing – original draft, Visualization, Validation, Resources, Project administration, Methodology, Investigation, Formal analysis, Data curation, Conceptualization. AL: Writing – original draft, Writing – review & editing, Data curation, Conceptualization. LH: Writing – review & editing, Writing – original draft, Conceptualization. DG: Writing – review & editing, Writing – original draft, Conceptualization. JG: Writing – review & editing, Writing – original draft, Methodology, Conceptualization. DW: Writing – review & editing, Writing – original draft, Methodology, Conceptualization. TH: Writing – review & editing, Writing – original draft. TE: Writing – review & editing, Writing – original draft, Visualization, Validation, Supervision, Resources, Project administration, Methodology, Investigation, Funding acquisition, Formal analysis, Data curation, Conceptualization.
