# Supplementary material for: Listening to the community: identifying obesity prevention strategies for rural preschool-aged children
Source: Front Public Health. 2024 May 31;12:1372890. doi: 10.3389/fpubh.2024.1372890 (PMC11177876; doi:10.3389/fpubh.2024.1372890)
Supplement: Supplementary file 1 [file Table_1.docx]

Supplementary Material

Listening to the community: Identifying obesity prevention strategies for rural preschool-aged children

Katherine Jochim Pope, RN, MPH, Alexandra F. Lightfoot, EdD, Lisa Macon Harrison, MPH, RD, Deborah Getz, ReD, Joel Gittelsohn, PhD, Dianne Ward, EdD, Tamara S. Hannon, MD, Temitope Erinosho, PhD

**Correspondence:** Dr. Temitope Erinosho: toerin@iu.edu

# Supplementary Figures and Tables

**Supplementary Table 1.** Questions that guided discussions with participants at the community workshops

| **Questions that Guided Discussions with Participants at the Community Workshops** |
| --- |
| - What is influencing the choice to provide [*insert behavioral target,* e.g., fewer fruits and vegetables] to children aged 2-5 years old at [*insert setting,* e.g., home]? |
| - What are some of the places where we could work in community to promote [*insert behavioral target*, e.g., more fruits and vegetables] with children aged 2-5 years and their families? |
| - Which of these places would you consider to be the most important to target in a health promotion program for your community? |
| - What are some things we can do in a health promotion program to promote [*insert behavioral target,* et., more fruits and vegetable intake] with children aged 2-5 years and families at [*insert setting,* e.g., home]? |
| - Which of these strategies would you consider to be the most important to prioritize in a health promotion program for your community? |
| - What are some challenges that we might encounter while implementing these health promotion strategies that you have prioritized? |
| - What are some things that we can do to address those challenges? |
| - Over the next couple of months, our team would like to work closely with you as we develop a health promotion program for children aged 2-5 years and their families. This would involve us having meetings with you to put the program together: |
| - Who in this room today wants to continue to be at the table and play a key role in developing the health promotion program? |
| - Who else should we invite to join us at the table? What organization do they represent? What is the best way to contact the person? |
| - How often should our group meet? When should we meet (day and time)? Where should our meetings take place? |
| - What can we do or put in place to motivate you to continue to stay actively engage group meetings? |
| - Which health promotion strategies from the list that you prioritized can we begin to implement together with little or no funding? |
| - What are potential funding opportunities that we can explore together to begin to do some of the work? |
| **Nutrition Behavioral Targets:** increase fruits and vegetables; decrease: fast food, sweet and salty snacks, and sugar-sweetened beverages. **Physical Activity Behavioral targets:** increase physical activity and reduce screen time. **Settings:** home; while eating out; day care; and other places where children spend time frequently. |
